# Patterns of Lymph Node Metastasis and Optimal Surgical Strategy in Small (≤20 mm) Gastroenteropancreatic Neuroendocrine Tumors

**DOI:** 10.3389/fendo.2022.871830

**Published:** 2022-07-21

**Authors:** Yibo Cai, Zhuo Liu, Lai Jiang, Dening Ma, Zhenyuan Zhou, Haixing Ju, Yuping Zhu

**Affiliations:** Department of Colorectal Surgery, The Cancer Hospital of the University of Chinese Academy of Sciences (Zhejiang Cancer Hospital), Institute of Basic Medicine and Cancer (IBMC), Chinese Academy of Sciences, Hangzhou, China

**Keywords:** gastroenteropancreatic neuroendocrine tumors, small tumor size, lymph node metastatic patterns, surgical strategy, SEER database

## Abstract

**Background:**

Regional lymph node metastasis (LNM) is crucial for planning additional lymphadenectomy, and is directly correlated with poor prognosis in gastroenteropancreatic neuroendocrine tumors (GEP-NETs). However, the patterns of LNM for small (≤20 mm) GEP-NETs remain unclear. This population-based study aimed at evaluating LNM patterns and identifying optimal surgical strategies from the standpoint of lymph node dissemination.

**Methods:**

This retrospective cohort study retrieved data from the Surveillance, Epidemiology, and End Results (SEER) 18 registries database for 17,308 patients diagnosed as having localized well-differentiated GEP-NETs ≤ 20 mm between January 1, 2004, and December 31, 2017. The patterns of LNM were characterized in 6,622 patients who underwent extended resection for adequate lymph node harvest.

**Results:**

Of 6,622 patients with localized small GEP-NETs in the current study, 2,380 (36%) presented with LNM after regional lymphadenectomy. Nodal involvement was observed in approximately 7.4%, 49.1%, 13.6%, 53.7%, 13.8%, 7.8%, and 15.4% of gastric (g-), small intestinal (si-), appendiceal (a-), colonic (c-), rectal (r-), non-functional pancreatic (nfp-), and functional pancreatic (fp-) NETs ≤ 20 mm. Patients with younger age, larger tumor size, and muscularis invasion were more likely to present with LNM. Additional lymphadenectomy conferred a significant survival advantage in NETs (≤10 mm: HR, 0.47; 95% CI, 0.33–0.66; *p* < 0.001; 11–20 mm: HR, 0.54; 95% CI, 0.34–0.85; *p* = 0.008) and fp-NETs ≤ 20 mm (HR, 0.08; 95% CI, 0.02–0.36; *p* = 0.001), as well as g-NETs (HR, 0.39; 95% CI, 0.16–0.96; *p* = 0.041) and c-NETs of 11–20 mm (HR, 0.07; 95% CI, 0.01–0.48; *p* = 0.007). Survival benefits of additional lymphadenectomy were not found in a-NETs, r-NETs, and nfp-NETs with a small size.

**Conclusions:**

Given the increased risk for nodal metastasis, primary tumor resection with regional lymphadenectomy is a potential optimal surgical strategy for si-NETs and fp-NETs ≤ 20 mm, as well as g-NETs and c-NETs of 11–20 mm. Local resection is an appropriate and reliable surgical approach for a-NETs, r-NETs, and nfp-NETs ≤ 20 mm.

## Introduction

Gastroenteropancreatic neuroendocrine tumors (GEP-NETs) are biologically heterogeneous neoplasms originating from neuroendocrine cells of the gastrointestinal tract and pancreas (1). Although they are relatively rare in clinical practice, the incidence of GEP-NETs has seen an incremental increase over the last three decades (2–4). Currently, surgical resection is the mainstay treatment for localized GEP-NETs. The tumor size influences decisions regarding the treatment choice for well-differentiated GEP-NETs. According to the current consensus, given the increased risk of nodal metastasis in GEP-NETs > 20 mm, tumor resection with regional lymphadenectomy is recommended as a surgical routine irrespective of the primary subsite (5). However, for GEP-NETs ≤ 20 mm, which are designated as small GEP-NETs, the optimal surgical strategy has yet to be determined. The inconclusive guidelines with regard to the surgical procedures are due to the ambiguity of nodal metastasis risk at different anatomical subsites. A limited number of studies have evaluated on the regional lymph node metastasis (LNM) patterns in small GEP-NETs with most of them assessing a single site of origin (6–12). Moreover, the impact of regional nodal involvement on prognosis in small GEP-NETs has not been conclusively determined. Blakely et al. (11) reported worse survival among patients with LNM in pancreatic NETs (p-NETs) ≤ 20 mm. However, this survival disadvantage was not observed in appendiceal NETs (a-NETs) (13). These discrepancies among studies provide inconclusive evidence for regional lymphadenectomy of small GEP-NETs, particularly in tumors of 10 to 20 mm. Given the heterogeneity and rarity of small well-differentiated GEP-NETs, we used a large national database to investigate LNM patterns in small GEP-NETs and to evaluate the benefits of regional lymphadenectomy, depending on the anatomical subsites and tumor size.

## Materials and Methods

### Data Source and Patient Selection

We used the Surveillance, Epidemiology, and End Results (SEER) database on the November 2019 submissions, which covers approximately 34% of the US population (14). Based on the International Classification of Disease for Oncology (ICD-O-3) histologic type (functional 8150–8157, non-functional 8240–8242) and topography codes (stomach C16.0–16.9, small intestine C17.0–17.9, appendix C18.1, colon C18.0 and 18.2–18.9, rectum C19.9 and 20.9, pancreas C25.0–25.9), the SEER 18 Registries database was queried to identify all eligible patients diagnosed as well-differentiated GEP-NETs from January 1, 2004 to December 31, 2017. Data on patient demographics (age at diagnosis, gender, race/ethnicity, and year of diagnosis) as well as tumor characteristics (tumor size and invasion, and nodal metastasis) were retrieved. All patients were histologically confirmed and required to have a tumor size measuring ≤ 20 mm. Tumor sizes were further dichotomized into ≤ 10 mm or 11–20 mm. Patients with distant or unstaged tumors were excluded from this study. According to the 8th edition of the American Joint Committee on Cancer (AJCC) TNM staging system, T category was determined by invasion depth and tumor size. Surgical interventions were collapsed into two categories based on surgical procedure codes. Patients subjected to local excision or enucleation (SEER site-specific surgery codes: 10-32 for a-NETs and 10-29 for other GEP-NETs) were designated as local resection (LR) cohort while those who underwent regional lymphadenectomy (SEER site-specific surgery codes: 40-80 for a-NETs and 30-80 for other GEP-NETs) were designated as the extended resection (ER) cohort. To accurately describe the presence of LNM in small GEP-NETs, we focused on individuals who underwent ER for adequate lymph node (LN) harvest.

### Statistical Analysis

Descriptive statistics were used to compare demographics and tumor characteristics across subgroups stratified by LN status. To accurately estimate survival outcomes, patients with multiple primary malignant tumors or those who had died within 30 days after confirmed diagnosis were excluded from survival analyses. Survival time was calculated from the date of diagnosis to the date of last follow-up or death. Estimates of survival probability were obtained by the Kaplan–Meier method and compared using the log-rank test. Multivariate adjusted Cox proportional hazards regression and binomial logistic regression models were fitted to evaluate covariates that were associated with overall mortality and LNM. In subgroup analysis, we investigated the efficacy of surgical interventions in relation to tumor location and size using a Cox proportional hazards model while adjusting for age at diagnosis, T category, and LN status. The SPSS statistical software version 22.0 (SPSS Inc., IBM Corporation, Chicago, IL) was used for statistical analyses. A two-tailed p-value ≤ 0.05 was set as the threshold for statistical significance.

## Results

### Characteristics of Small GEP-NETs That Underwent Regional Lymphadenectomy Stratified by LN Status

We identified 17,308 patients diagnosed as having localized small well-differentiated GEP-NETs, including 6,622 patients who underwent regional lymphadenectomy (ER cohort). Demographics and tumor characteristics of the ER cohort stratified by LN status are summarized in **Table 1**. A total of 2,380 patients (36.0%) had LNM in the ER cohort. The association between age at diagnosis and LNM was significant (p < 0.001). A high risk of LNM was highly associated with small intestinal NETs (si-NETs) (49.1%) and colonic NETs (c-NETs) (53.7%) patients. The LNM proportions for gastric NETs (g-NETs), a-NETs, rectal NETs (r-NETs), non-functional NETs (nfp-NETs), and functional pancreatic NETs (fp-NETs) were 7.4%, 13.6%, 13.8%, 7.8%, and 15.4%, respectively. Substantial differences by T category were found in the LNM risk (p < 0.001). The LNM rate increased from 12.5% (T1) to 45.9% (T2), 59.4% (T3), and 68.3% (T4). Compared to tumors of ≤10 mm, the prevalence of LNM was twofold higher in tumors of 11–20 mm (20.0% vs 49.8%).

**Table 1:** Demographics of patients stratified by LN status and clinicopathologic characteristics associated with LN metastasis.

Characteristic	Patients, No. (%)		*p-*value	OR (95% CI)	*p-*value
	LN positive	LN negative			
No.	2,380 (36.3)	4,182 (63.7)		–
Age at diagnosis, years			0.001	
≤50	438 (32.1)	928 (67.9)		1 [Reference]	
51–70	1,308 (37.4)	2,185 (62.6)		1.04 (0.88–1.22)	0.665
>70	634 (37.2)	1,069 (62.8)		0.73 (0.61–0.88)	0.001
Sex			0.795	
Male	1,182 (36.4)	2,063 (63.6)		–
Female	1,198 (36.1)	2,119 (63.9)		
Race			<0.001	
White	1,974 (37.8)	3,246 (62.2)		1 [Reference]	
Black	314 (32.4)	655 (67.6)		0.78 (0.66–0.92)	0.004
Asian/PI/AI	79 (24.2)	247 (75.8)		0.98 (0.71–1.34)	0.885
Unknown	13 (27.7)	34 (72.3)		0.89 (0.40–2.00)	0.778
Year of diagnosis			<0.001		
2004–2008	700 (41.4)	992 (58.6)		1 [Reference]	
2009–2013	742 (36.0)	1,320 (64.0)		1.05 (0.86–1.28)	0.649
2013–2017	938 (33.4)	1,870 (66.6)		1.42 (1.14–1.77)	0.002
First primary malignancy			0.032		
Yes	536 (34.0)	1,040 (66.0)		0.94 (0.84–1.11)	0.617
No	1,844 (37.0)	3,142 (73.0)		1 [Reference]	
Primary site			<0.001		<0.001
Stomach	31 (7.4)	388 (92.6)		1 [Reference]	
Small intestine	1,940 (49.1)	2,015 (50.9)		10.36 (7.06–15.22)	<0.001
Appendix	59 (13.6)	374 (86.4)		2.81 (1.73–4.57)	<0.001
Colon	212 (53.7)	183 (46.3)		14.77 (9.55–22.83)	<0.001
Rectum	51 (13.8)	319 (86.2)		3.51 (2.14–5.76)	<0.001
Non-functional pancreas)	67 (7.8)	793 (92.2)		1.09 (0.68-1.76)	0.724
Functional pancreas	20 (15.4)	110 (84.6)		2.57 (1.36–4.86)	<0.001
T category			<0.001		
T1	307 (12.5)	2,153 (87.5)		1 [Reference]	
T2	432 (45.9)	509 (54.1)		1.84 (1.47–2.30)	<0.001
T3	704 (59.4)	482 (40.6)		2.87 (2.32–3.54)	<0.001
T4	254 (68.3)	118 (31.7)		3.41 (2.54–4.57)	<0.001
Unknown	683 (42.6)	920 (57.4)		2.29 (1.78–2.95)	<0.001
Tumor size			<0.001		
≤10 mm	569 (20.0)	2,386 (80.0)		1 [Reference]	
11–20 mm	1,784 (49.8)	1,796 (50.2)		3.38 (2.95–3.88)	<0.001

GEP-NETs, gastroenteropancreatic neuroendocrine tumors; LN, lymph node; SEER, Surveillance, Epidemiology, and End Results; OR, odds ratio; CI, confidence interval; PI/AI, Pacific Islander/American Indian.

### Patterns and Predictive Factors of LNM in Small Well-Differentiated GEP-NETs

In the ER cohort, LNM was associated with poor cancer-specific survival (CSS) in small GEP-NETs [hazard ratio (HR), 1.58; 95% CI, 1.13–2.19; p = 0.007] (**Supplementary Figure S1**). Younger age, tumors originating from small intestine and colon, deeper tumor invasion, and larger tumor size were associated with the likelihood of LNM (**Table 1**). Compared to younger patients, patients older than 70 years were less likely to have LNM [odds ratio (OR), 0.73; 95% CI, 0.61–0.88; p = 0.001]. This inverse association between age and LNM was found in the g-NETs, si-NETs, and a-NETs (**Table 2**). The abundance of LNM for g-NETs, si-NETs, a-NETs, c-NETs, r-NETs, nfp-NETs, and fp-NETs ≤ 10 mm was 4.1%, 28.9%, 4.0%, 24.9%, 6.8%, 7.2% and 9.7%. The corresponding prevalence of LNM for tumors of 11–20 mm was 12.1%, 64.1%, 29.8%, 76.1%, 40.3%, 8.1%, and 17.2%. In the T category, 307 T1 patients (12.4%) had LNM. GEP-NETs in Category T1 originated from small intestine (22.3%) and colon (28.3%), while those in category T2–T4 (55.1%) were associated with a high risk of LNM. For g-NETs, a-NETs, r-NETs, and nfp-NETs, LNM rates were reduced to less than 6% in specific individuals with category T1 and lesions ≤ 10 mm.

**Table 2:** Patterns of LNM in small well-differentiated GEP-NETs receiving regional lymphadenectomy.

Characteristic	Patients with LNM (underwent with ER), No. (%) ^a^									
	Stomach		Small intestine		Appendix		Colon	Rectum	Non-functional pancreas	Functional pancreas
Total		31 (7.4)		1,940 (48.4)	59 (13.6)	212 (53.7)	51 (13.8)		67 (7.8)	20 (15.4)
Age										
≤50		10 (8.7)		318 (49.5)	42 (21.0)	34 (38.2)	13 (12.7)		12 (7.0)	9 (19.1)
51–70		20 (7.8)		1,067 (51.5)	15 (8.0)	156 (58.6)	36 (14.2)		51 (8.2)	8 (12.3)
>70		1 (2.1)		555 (44.7)	2 (4.4)	22 (55.0)	2 (13.3)		4 (6.2)	3 (16.7)
Tumor size										
≤10 mm		10 (4.1)		489 (28.9)	11 (4.0)	43 (24.9)	20 (6.8)		20 (7.2)	3 (9.7)
11–20 mm		21 (12.1)		1,451 (64.1)	48 (29.8)	169 (76.1)	31 (40.3)		47 (8.1)	17 (17.2)
T category										
T1		9 (5.5)		150 (22.4)	36 (9.7)	32 (28.3)	22 (9.2)		46 (5.8)	12 (10.3)
T2–T4		12 (9.2)		1,230 (58.6)	7 (36.8)	101 (73.2)	12 (44.4)		20 (30.3)	8 (66.7)
Tumor size + T category										
T1+ ≤ 10 mm		9 (5.7)		150 (22.5)	7 (2.8)	20 (22.0)	10 (5.0)		16 (6.0)	3 (10.0)
T1+ 11-20 mm		0 (0.0)		0 (0.0)	29 (24.4)	12 (54.5)	12 (30.8)		30 (5.7)	9 (10.5)
T2–T4+ ≤ 10 mm		1 (7.7)		186 (36.4)	2 (40.0)	9 (39.1)	3 (25.0)		4 (36.4)	0 (0.0)
T2–T4+ 11-20 mm		11 (8.9)		1,044 (65.5)	5 (35.7)	92 (80.0)	9 (60.0)		16 (29.1)	8 (72.7)

LNM, Lymph Node Metastasis; GEP-NETs, Gastroenteropancreatic Neuroendocrine Tumors; ER, Extended Resection. ^a^ The rate of lymph node metastasis in small well-differentiated GEPNET was observed in cases without distant metastases and underwent extended resection for the adequate lymph node harvest.

### The Impact of LNM on Prognosis and the Optimal Surgical Strategy for Small Well-Differentiated GEP-NETs

**Figure 1:**
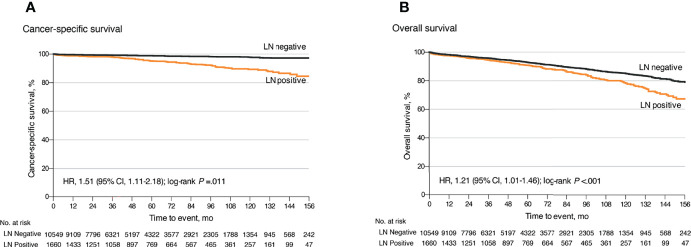
Kaplan–Meier curves for cancer-specific and overall survival for all patients with small well-differentiated GEP-NETs stratified by LN status. **(A)** Kaplan–Meier analysis of cancer-specific survival. **(B)** Kaplan–Meier analysis of overall survival. GEP-NETs, gastroenteropancreatic neuroendocrine tumors; LN, lymph node.

**Figure 2:**
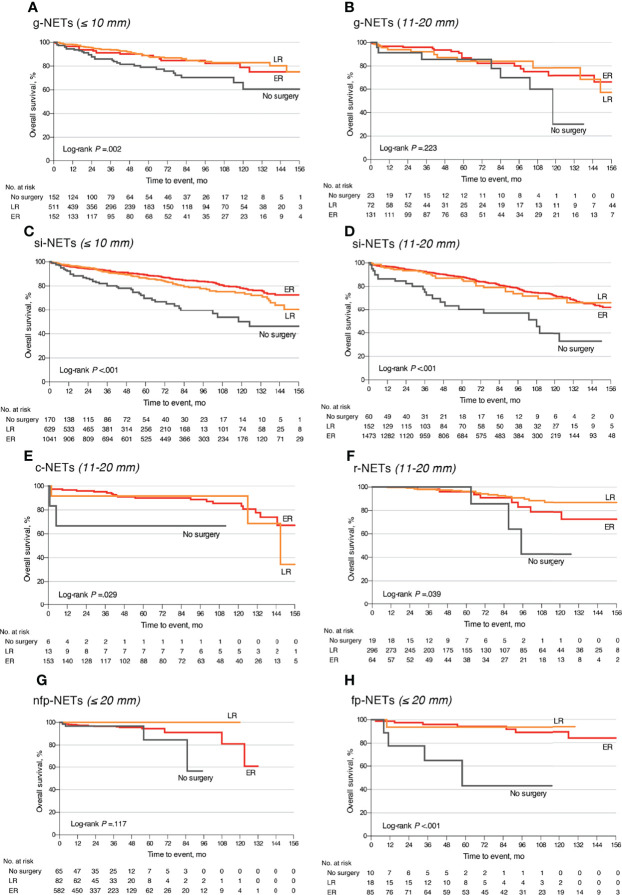
Overall survival comparison of different surgical strategies stratified by tumor location and size among patients with small well-differentiated GEP-NETs. **(A)** The g-NETs of the ≤10 mm cohort; **(B)** the g-NETs of the 11–20 mm cohort; **(C)** the si-NETs of the ≤10 mm cohort; **(D)** the si-NETs of the 11–20 mm cohort; **(E)** the c-NETs of the 11–20 mm cohort; **(F)** the r-NETs of the 11–20 mm cohort; **(G)** the nfp-NETs of the ≤20 mm cohort; and **(H)** the fp-NETs of the ≤20 mm cohort. GEP-NETs, gastroenteropancreatic neuroendocrine tumors; LR, local resection; ER, extended resection; g-NETs, gastric neuroendocrine tumors; si-NETs, small intestinal neuroendocrine tumors; c-NETs, colonic neuroendocrine tumors; r-NETs, rectal neuroendocrine tumors; nfp-NETs, non-functional pancreatic neuroendocrine tumors; fp-NET, functional pancreatic neuroendocrine tumors.

Among the 17,308 small GEP-NETs cases, the 10-year CSS and overall survival (OS) were 96.4% and 84.0%, respectively. LNM was established to be an independent predictor for poor CSS (HR, 1.55; 95% CI, 1.11–2.18; p = 0.011) and OS (HR, 1.21; 95% CI, 1.01–1.46; p = 0.041) (**Figures 1A, B**). Compared to the observation strategy, LR and ER reduced the risk of cancer-specific mortality by 57% and 35% (p = 0.003 and p = 0.095) and all-cause mortality by 42% and 41% (p < 0.001 for both), respectively (**Supplementary Table S1**). In subgroup analysis, LR prolonged the 10-year OS for g-NETs (HR, 0.55; 95% CI, 0.35–0.86; p = 0.009; **Figure 2A**) and si-NETs ≤ 10 mm (HR, 0.53; 95% CI, 0.37–0.76; p < 0.001; **Figure 2C**), whereas this survival benefit was not apparent in other GEP-NETs ≤ 10 mm (**Table 3**). Moreover, ER significantly improved OS for si-NETs ≤ 10 mm (HR, 0.47; 95% CI, 0.33–0.66; p < 0.001) but not for g-NETs (HR, 0.81; p = 0.475). In the 11–20 mm cohort, compared to the observation strategy, ER exhibited favorable OS for g-NETs (HR, 0.39; 95% CI, 0.16–0.96; p = 0.041; **Figure 2B**), si-NETs (HR, 0.54; 95% CI, 0.34–0.85; p = 0.008; **Figure 2D**), and c-NETs (HR, 0.07; 95% CI, 0.01–0.48; p = 0.007; **Figure 2E**), whereas this survival advantage was not observed in a-NETs (HR, 6.93; p = 0.092). LR was effective in prolonging OS in r-NET (HR, 0.25; 95% CI, 0.07–0.86; p = 0.028; **Figure 2F**); however, there were no apparent survival benefits associated with regional lymphadenectomy (HR, 0.38; p = 0.183). Majority of patients with fp-NETs and nfp-NETs ≤ 20 mm underwent ER, which achieved an excellent 10-year OS rates of 81.0% and 81.9%, respectively. Our results imply that extended removal can significantly improve long-term survival for fp-NETs ≤ 20 mm patients (HR, 0.08; 95% CI, 0.02–0.36; p = 0.001; **Figure 2H**) but not for the non-functional ones (HR, 1.33; p = 0.694; **Figure 2G**).

**Table 3:** Comparisons of different surgical managements stratified by tumor location and size in small well-differentiated GEP-NETs.

Characteristic	Univariate (10-year OS)					Multivariate (HR, 95% CI)					
	Observation	LR	ER		*p*	LR (vs. Observation)	*p*		ER (vs. Observation)	*p*
**≤10 mm**											
Stomach	0.605	0.827	0.788		0.002	0.55 (0.35–0.86)	0.009		0.81 (0.45–1.45)	0.475	
Small intestine	0.500	0.748	0.785		<0.001	0.53 (0.37–0.76)	<0.001		0.47 (0.33–0.66)	<0.001	
Appendix	NA^a^	0.974	0.986		0.975	1 [Reference]	– ^b^		0.56 (0.12–2.57)	0.457	
Colon	0.854	0.892	0.799		0.025	0.65 (0.19–2.27)	0.499		1.39 (0.38–5.02)	0.619	
Rectum	0.919	0.924	0.948		0.380	0.80 (0.49–1.32)	0.382		0.47 (0.16–1.40)	0.176	
**11**–**20 mm**											
Stomach	0.300	0.772	0.718		0.230	0.53 (0.20–1.38)	0.192		0.39 (0.16–0.96)	0.041	
Small intestine	0.406	0.688	0.724		<0.001	0.61 (0.35–1.07)	0.085		0.54 (0.34–0.85)	0.008	
Appendix	NA^a^	0.996	0.941		0.157	1 [Reference]	– ^b^		6.93 (0.73–65.80)	0.092	
Colon	0.667	0.688	0.855		0.029	0.13 (0.02–0.97)	0.046		0.07 (0.01–0.48)	0.007	
Rectum	0.429	0.869	0.789		0.042	0.25 (0.07–0.86)	0.028		0.38 (0.09–1.58)	0.183	
**≤20 mm**											
Non-functional pancreas	0.564	1.00	0.810		0.117	NA^c^			1.33 (0.32–5.44)	0.694	
Functional pancreas	0.432	0.938	0.893		<0.001	0.39 (0.04–3.86)	0.420		0.08 (0.02–0.36)	0.001	

GEP-NETs, gastroenteropancreatic neuroendocrine tumors; OS, overall survival; HR, hazard ratio; CI, confidence interval; LR, local resection; ER, extended resection; NA, not applicable. ^a^ Estimates were not calculated when the number of cases per subgroup was less than 10. ^b^ The HR was estimated with a Cox regression analysis using the LR group as the reference, due to the small sample of observation group. ^c^ The limited number of events was not provided enough statistical power to fit the Cox regression model.

## Discussion

LN involvement, a critical predictor for poor survival, is rarely encountered in well-differentiated GEP-NETs measuring ≤ 20 mm (15, 16). In our ER cohort, a significantly increased cancer-specific mortality rate was also observed after histologically proven LNM (HR, 1.58; 95% CI, 1.13–2.19; p = 0.007). Therefore, it is important to elucidate the actual LNM patterns in small GEP-NETs. We designed this first and largest population-based study with long-term follow-up, to accurately describe the actual distribution of LNM and evaluate the equivocal survival benefits from regional lymphadenectomy in small GEP-NETs.

Well-differentiated NETs from the stomach are incidentally detected during gastroscopy and frequently manifest with small size (≤20 mm) (17). Most of the small well-differentiated tumors belong to type 1 g-NETs (17–19). Based on the European Neuroendocrine Tumor Society (ENETS) guidelines, for this type with superficial and low-grade features, endoscopic resection and surveillance are considered adequate because of the low tumor-related mortality and acceptable LNM rates (less than 2%–9%) (4, 17, 18). Herein, the LNM rate was 4.1% for g-NETs ≤ 10 mm. However, for tumors of 11–20 mm, there was a remarkable increase in the LNM rate, which reached 12.1%, with previous studies reporting a range of 8.6 to 15.3% (6, 20, 21). Consistent with a recent study (22), we found that LNM was slightly associated with unfavorable OS in small g-NETs (HR, 2.48; p = 0.061) and regional lymphadenectomy had survival advantages for g-NETs 11–20 mm (HR, 0.39; 95% CI, 0.16–0.96; p = 0.041). In Japan, lymphadenectomy has been suggested for g-NET 11–20 mm. Given the non-negligible risk of LNM, it seems to be a more appropriate surgical strategy (17, 19).

The small intestine is a common site for GEP-NETs (4, 23). However, si-NETs are unique malignancies with poor prognosis. Despite their small sizes, si-NETs frequently present with LNM and approximately 46%–98% of patients have nodal involvement after regional lymphadenectomy (23, 24). Herein, LN positivity was pathologically detected in 49.1% of si-NETs ≤ 20 mm. Given this high propensity for regional lymphatic spread, mesenteric lymphadenectomy during small bowel resection is recommended by the ENETS guideline (25). Our findings and those of previous studies further substantiated this recommendation, given that si-NETs that underwent lymphadenectomy exhibited improved survival, relative to those receiving LR only, irrespective of tumor size (26).

Owing to the heterogeneous nature of GEP-NETs, the malignant potential of a-NETs and r-NETs differs from those derived from the small intestines (4). A-NETs and r-NETs ≤ 10 mm are less likely to present with nodal involvement. In previous studies, only one patient (3.8%) with a-NETs ≤ 10 mm harbored occult LNM, and 9.2% of G1/2 r-NETs ≤ 10 mm, which were 4.0% and 6.8% herein (7, 27). Thus, sample appendectomy or endoscopic resection are the mainstay treatment for a-NETs and r-NETs ≤ 10 mm (7, 28). Our data revealed excellent long-term survival of a-NETs and r-NETs ≤ 10 mm underwent LR, with 10-year OS rates of 97.4% and 92.4%, respectively. However, for tumors 11–20 mm, the optimal surgical strategy remains elusive. Some investigators suggested that, because of the indolent nature, appendectomy and complete endoscopic resection are sufficient for a-NETs and r-NETs of 11–20 mm (5, 8, 28), whereas other investigators are prudent because of the elevated risk of LNM in these patients, which were also observed in our study (a-NETs, 29.8%; r-NETs, 40.3%) (9, 27, 29). A tumor size of 11–20 mm is identified as a predictor for LNM in a-NETs (OR, 5.5; p < 0.001) and r-NETs (OR, 4.24; p < 0.01) (9). These findings do not demonstrate that additional lymphadenectomy is worthwhile for tumors of 11–20 mm. Herein, radical resection did not appear to improve OS, as has been previously reported (8, 10). This discrepancy might be attributed to the increased morbidity and decreased quality of life after radical resection. Therefore, additional lymphadenectomy should be cautiously recommended for a-NETs and r-NETs of 11–20 mm. The treatment algorithm based solely on tumor size is insufficient and ambiguous. We believe that lymphadenectomy is warranted for tumors of 11–20 mm with predictors of aggressive tumor behaviors, such as grade 2-3, muscularis, or lymphovascular invasion (7, 9, 30).

C-NETs are always large tumors, and small lesions are rarely encountered during colonoscopy. They tend to behave aggressively and have a high metastatic potential for LN (4). The National Comprehensive Cancer Network (NCCN) guidelines recommend bowel resection with regional lymphadenectomy, irrespective of tumor size; however, the standard surgical strategy for small c-NETs has not been clearly elucidated (31). We confirmed a high risk of LNM in small c-NETs. LNM occurred in 24.9% of tumors ≤ 10 mm and 76.1% of tumors 11–20 mm. Comparable findings reported by Natour et al. (32) were 26.9% in c-NETs ≤ 10 mm and 65.1% in 11–20 mm after LN sampling. Bowel resection with lymphadenectomy remained the mainstay treatment in our 11–20 mm cohort, and was shown to improve OS (HR, 0.07; 95% CI, 0.01–0.48; p = 0.007). However, for c-NETs ≤ 10 mm, this survival benefit could not be observed after ER. In an analysis of nationwide registers in Japan, Konishi et al. (33) considered that c-NETs ≤ 10 mm without lymphatic invasion could be curatively treated by LR. The optimal treatment strategy for tumor ≤ 10 mm warrants further investigation.

In the last 4 decades, incidences of small p-NETs (≤20 mm) have shown a threefold increase, which could be accounted for by the widespread use of morphological and functional imaging (12). However, due to the uncertain malignant potential for small p-NETs, treatment algorithms remain controversial according to the current guidelines (31, 34). For asymptomatic nfp-NETs ≤ 20 mm, ENETS recommends an active surveillance policy considering the comparable safety of this policy and non-negligible complications of aggressive surgery (34). A recent meta-analysis reported the presence of LNM in 11.2% of small nfp-NETs, which was associated with worse prognosis. Thus, the surveillance policy was challenged and additional lymphadenectomy was suggested by these investigators (35). We found that 7.8% of nfp-NETs ≤ 20 mm patients had occult LNM, in tandem with previous findings from a German study (36). Notably, patients with nfp-NETs ≤ 20 mm who received local enucleation exhibited excellent prognosis after 10 years’ follow-up herein (10-year OS = 100%), compared to 56.4% in the observation group (p = 0.117). Due to the potential selection bias, this reduced OS in the observation arm should be interpreted with caution. The impact of formal lymphadenectomy on survival was not found, which could be attributed to inadequate LN harvest and increased perioperative mortality (12). For fp-NETs ≤ 20 mm, according to the NCCN guidelines, enucleation with peripancreatic LN dissection is recommended, except for insulinomas with a benign course (31). Pancreatic gastrinomas, VIPomas, and glucagonomas harbor malignant potential and are associated with LNM (35, 37–39). Herein, LNM was found in 15.4% of all fp-NETs ≤ 20 mm. The possible explanation for the different LNM patterns between small fp-NETs and nfp-NETs may be the tumor heterogeneity and metastatic potential are enhanced in patients with fp-NETs. However, the underlying mechanisms remain unclear. In tandem with previous findings (40), LNM exhibited a negative effect on survival (HR, 9.84; p = 0.005). Peripancreatic LN removal should be considered as it improved survival (HR, 0.08; 95% CI, 0.02–0.36; p = 0.001). Therapeutic decisions should be made by a multidisciplinary team, especially when surveillance policies or extended surgeries are discussed, to carefully weigh the benefits against the risk for each small p-NET (41).

Our results must be considered in light of several limitations. First, information regarding factors that might predispose one to nodal metastasis and recurrence, such as Ki-67 index, mitotic rate, vertical margin, lymphatic and blood vascular invasion, and genetic backgrounds, is unavailable in the SEER database. Therefore, we were unable to evaluate the association between these pathological parameters and LNM. Another intrinsic limitation is non-negligible variability in the LN yield for each GEP-NET, reflecting real-world clinical settings. Currently, there are no guidelines to make an explicit statement of the optimal number of examined LNs, and most surgeons implement lymphadenectomy based on clinical experience. Our study focuses on patients who underwent ER with the hope of reflecting the actual patterns of regional LNM in small GEP-NETs. In this study, a proportion of patients without examined LNs were regarded as those without LNM, which may have been caused by inadequate LN harvest, resulting in an increased false LN negative rate.

## Conclusions

Among localized small GEP-NETs that underwent ER, 2,380 patients (36.0%) were identified as LNM. GEP-NETs with younger age, derived from small intestine or colon, with a tumor size of 11 to 20 mm, and with deeper tumor invasion were more likely to present with LNM. Given the increased prevalence of regional nodal metastasis, tumor resection with lymphadenectomy seemed to be the optimal surgical strategy for si-NETs and fp-NETs ≤ 20 mm, as well as g-NETs and c-NETs of 11–20 mm. Survival benefits of additional lymphadenectomy were not found in a-NETs, r-NETs, and nfp-NETs with a small size.

## Data Availability Statement

The datasets presented in this study can be found in online repositories. The names of the repository/repositories can be found in the article/**Supplementary Material**. The accession number of SEER 18 registries database is 11713-Nov2019.

## Author Contributions

YZ, HJ, and YC contributed to the conception and design of the study. YC, ZL, and LJ collected data and organized the database. YC, DM, and ZZ performed the statistical analysis. YZ, HJ, YC, ZL, and LJ interpreted the results. YC and YZ wrote the first draft of the manuscript. YC, YZ, and HJ wrote sections of the manuscript. All authors contributed to manuscript revision, read, and approved the submitted version.

## Funding

This study is supported in part by the Medical and Health Research Project of Zhejiang Province (grant 2020KY468) and the Natural Science Foundation of Zhejiang Province (grant LY20H160001).

## Conflict of Interest

The authors declare that the research was conducted in the absence of any commercial or financial relationships that could be construed as a potential conflict of interest.

## Publisher’s Note

All claims expressed in this article are solely those of the authors and do not necessarily represent those of their affiliated organizations, or those of the publisher, the editors and the reviewers. Any product that may be evaluated in this article, or claim that may be made by its manufacturer, is not guaranteed or endorsed by the publisher.
