# Supplementary material for: Patterns of Lymph Node Metastasis and Optimal Surgical Strategy in Small (≤20 mm) Gastroenteropancreatic Neuroendocrine Tumors
Source: Front Endocrinol (Lausanne). 2022 Jul 21;13:871830. doi: 10.3389/fendo.2022.871830 (PMC9350735; doi:10.3389/fendo.2022.871830)
Supplement: Supplementary file 1 [file Image_1.pdf]

Supplementary Material

Supplementary Figures

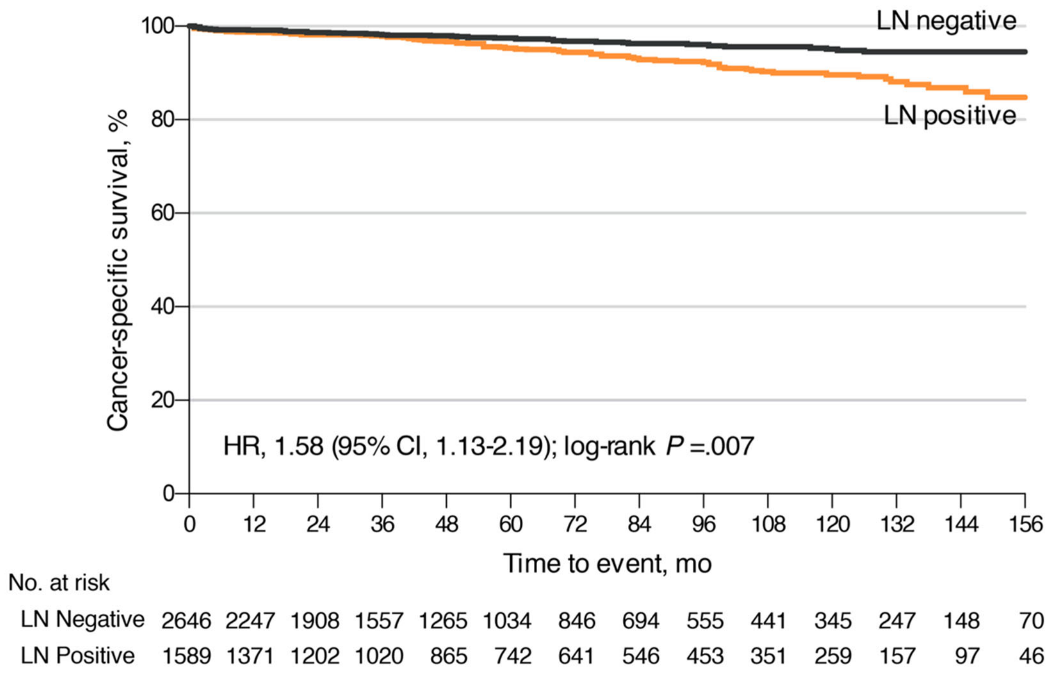

Supplementary Figure S1. Kaplan–Meier curves for cancer-specific survival stratified by LN status in small well-differentiated GEP-NETs receiving regional lymphadenectomy. LN, lymph node; GEP-NETs, gastroenteropancreatic neuroendocrine tumors.
