# Supplementary material for: Patterns of Lymph Node Metastasis and Optimal Surgical Strategy in Small (≤20 mm) Gastroenteropancreatic Neuroendocrine Tumors
Source: Front Endocrinol (Lausanne). 2022 Jul 21;13:871830. doi: 10.3389/fendo.2022.871830 (PMC9350735; doi:10.3389/fendo.2022.871830)
Supplement: Supplementary file 2 [file Table_1.docx]

| **TABLE S1 \|** Prognostic factors associated with survival in small well-differentiated GEP-NETs underwent regional lymphadenectomy. | | | | | | |
| --- | --- | --- | --- | --- | --- | --- |
| **Characteristic** | **10-year CSS** | |  | **10-year OS** | | |
|  | **HR (95% CI)** | ***P*** |  | **HR (95% CI)** | ***P*** | |
| Age |  |  | |  |  | |
| **≤** 50 | 1 [Reference] |  | | 1 [Reference] | |  |
| 51-70 | 1.93 (1.23-3.03) | 0.004 | | 2.88 (2.29-3.63) | | <0.001 |
| > 70 | 6.62 (4.20-10.42) | <0.001 | | 10.40 (8.22-13.15) | | <0.001 |
| Race |  |  | |  | |  |
| White | 1 [Reference] |  | | 1 [Reference] | |  |
| Black | 1.18 (0.81-1.70) | 0.391 | | 1.54 (1.33-1.79) | | <0.001 |
| Asian/PI/AI | 0.84 (0.44-1.62) | 0.599 | | 0.74 (0.55-0.98) | | 0.037 |
| Primary sites |  |  | |  | |  |
| Stomach | 1 [Reference] |  | | 1 [Reference]) | |  |
| Small intestine | 1.46 (0.81-2.61) | 0.208 | | 1.07 (0.87-1.31) | | 0.510 |
| Appendix | 1.12 (0.43-2.93) | 0.813 | | 0.45(0.28-0.72) | | 0.001 |
| Colon | 1.33 (0.63-2.78) | 0.458 | | 0.83 (0.61-1.14) | | 0.246 |
| Rectum | 0.49 (0.24-1.00) | 0.050 | | 0.43 (0.34-0.54) | | <0.001 |
| Non-functional Pancreas | 1.54 (0.66-3.58) | 0.322 | | 0.52 (0.33-0.80) | | 0.003 |
| Functional Pancreas | 1.88 (0.68-5.25) | 0.226 | | 0.65 (0.35-1.20) | | 0.168 |
| T category |  |  | |  | |  |
| T1 | 1 [Reference] |  | | 1 [Reference] | |  |
| T2-4 | 0.98 (0.58-1.63) | 0.930 | | 1.01 (0.82-1.26) | | 0.906 |
| LN metastasis |  |  | |  | |  |
| Negative | 1 [Reference] |  | | 1 [Reference] | |  |
| Positive | 1.55 (1.11-2.18) | 0.011 | | 1.21 (1.01-1.46) | | 0.041 |
| Tumor size |  |  | |  | |  |
| ≤ 10 mm | 1 [Reference] |  | | 1 [Reference] | |  |
| 11-20 mm | 2.52 (1.76-3.60) | <0.001 | | 1.29 (1.10-1.52) | | 0.002 |
| Surgical procedures |  |  | |  | |  |
| Observation | 1 [Reference] |  | | 1 [Reference] | |  |
| LR | 0.43 (0.25-0.76) | 0.003 | | 0.58 (0.48-0.71) | | <0.001 |
| ER | 0.65 (0.39-1.08) | 0.095 | | 0.59 (0.48-0.73) | | <0.001 |
| *GEP-NETs, gastroenteropancreatic neuroendocrine tumors; CSS, cancer-special survival; OS, overall survival; HR, hazard ratio; CI, confidence interval; PI/AI, Pacific Islander/American Indian; LR, local resection; ER, extended resection.* | | | | | | |
